# Quantitative susceptibility mapping and blood neurofilament light chain differentiate between parkinsonian disorders

**DOI:** 10.3389/fnagi.2022.909552

**Published:** 2022-08-05

**Authors:** Piao Zhang, Junling Chen, Tongtong Cai, Chentao He, Yan Li, Xiaohong Li, Zhenzhen Chen, Lijuan Wang, Yuhu Zhang

**Affiliations:** ^1^The Second School of Clinical Medicine, Southern Medical University, Guangzhou, China; ^2^Department of Neurology, Guangdong Neuroscience Institute, Guangdong Provincial People’s Hospital, Guangdong Academy of Medical Sciences, Guangzhou, China; ^3^Department of Neurology, Shantou Central Hospital, Shantou, China

**Keywords:** quantitative susceptibility mapping, iron deposition, neurofilament light chain, Parkinson’s disease, multiple system atrophy, progressive supranuclear palsy

## Abstract

**Objectives:**

We employed quantitative susceptibility mapping (QSM) to assess iron deposition in parkinsonian disorders and explored whether combining QSM values and neurofilament light (NfL) chain levels can improve the accuracy of distinguishing Parkinson’s disease (PD) from multiple system atrophy (MSA) and progressive supranuclear palsy (PSP).

**Materials and methods:**

Forty-seven patients with PD, 28 patients with MSA, 18 patients with PSP, and 28 healthy controls (HC) were enrolled, and QSM data were reconstructed. Susceptibility values in the bilateral globus pallidus (GP), putamen (PUT), caudate nucleus (CN), red nucleus (RN), substantia nigra (SN), and dentate nucleus (DN) were obtained. Plasma NfL levels of 47 PD, 18 MSA, and 14 PSP patients and 22 HC were measured by ultrasensitive Simoa technology.

**Results:**

The highest diagnostic accuracy distinguishing MSA from PD patients was observed with increased susceptibility values in CN (AUC: 0.740). The susceptibility values in RN yielded the highest diagnostic performance for distinguishing PSP from PD patients (AUC: 0.829). Plasma NfL levels were significantly higher in the MSA and PSP groups than in PD and HC groups. Combining the susceptibility values in the RN and plasma NfL levels improved the diagnostic performance for PSP vs. PD (AUC: 0.904), whereas plasma NfL levels had higher diagnostic accuracy for MSA vs. PD (AUC: 0.877).

**Conclusion:**

The exploratory study indicates different patterns of iron accumulation in deep gray matter nuclei in Parkinsonian disorders. Combining QSM values with NfL levels may be a promising biomarker for distinguishing PSP from PD, whereas plasma NfL may be a reliable biomarker for differentiating MSA from PD. QSM and NfL measures appeared to have low accuracy for separating PD from controls.

## Introduction

The differential diagnosis between Parkinson’s disease (PD) and atypical parkinsonian disorders (APDs), especially multiple system atrophy (MSA) and progressive supranuclear palsy (PSP), remains difficult in the early stages (Rizzo et al., 2016). There are no proven specific biomarkers, which means that autopsy pathologic analysis remains the gold standard for confirming clinical diagnosis and this situation is not conducive to treatment and prognosis (Jabbari et al., 2020). Given this challenge, defining disease-specific biomarkers to improve diagnostic accuracy is urgently needed.

It has been shown that abnormal metabolism of brain iron results in excessive iron deposition in PD, MSA, and PSP, which may result in neuronal degeneration and the occurrence of typical clinicopathological lesions associated with neurodegenerative disease. Previous studies using susceptibility weighted imaging (SWI) and R2* mapping to assess iron content have shown different iron deposition patterns in Parkinsonian disorders (Han et al., 2013; Lee et al., 2015; Langley et al., 2017). Quantitative susceptibility mapping (QSM) is believed to be distinctly more sensitive than SWI and R2* mapping for the quantitative analysis of iron content in the deep gray matter nuclei, and validated by a postmortem validation study (Langkammer et al., 2012). Previous QSM studies in APDs have found more iron content in the substantia nigra (SN) of PD patients, higher iron burden in the putamen (PUT) or red nucleus (RN) of MSA patients, and the most iron content in the RN or globus pallidus (GP) of PSP patients (Sjöström et al., 2017; Azuma et al., 2019; Mazzucchi et al., 2019). However, these studies did not take into account the role of susceptibility values measured on QSM in the caudate nucleus (CN) and dentate nucleus (DN) for distinguishing parkinsonian disorders (Sjöström et al., 2017; Azuma et al., 2019; Mazzucchi et al., 2019). In addition, the susceptibility values of QSM provides high specificity but relatively low sensitivity for differentiating between parkinsonian disorders with high accuracy (Azuma et al., 2019).

Until now, there are no other cerebrospinal fluid (CSF) or blood biomarkers that can potentially discriminate PD from MSA and PSP (Aerts et al., 2012; Chahine et al., 2014), with the exception of neurofilament light (NfL) chain (Hansson et al., 2017). NfL, highly expressed in large-caliber myelinated axons and involved in axonal growth and regeneration, is a marker of axonal degeneration (Petzold, 2005). Invasive lumbar puncture is not easily implemented because of patients’ anxiety about its complications, but blood NfL strongly correlated with CSF NfL (Hansson et al., 2017). Therefore, blood NfL may be a promising biomarker for APDs. Previous studies have shown that NfL in blood can distinguish between PD and APDs (e.g., PSP and MSA), with a high degree of diagnostic accuracy (Hansson et al., 2017; Jabbari et al., 2020). However, NfL in blood and CSF cannot yet discriminate between MSA and PSP (Hansson et al., 2017); simultaneously, NfL is not a disease-specific biomarker, and its specificity is relatively low with increased NfL levels in other nervous system diseases (Palermo et al., 2020).

Therefore, it is possible that combining QSM and assessment of NfL could provide a biomarker for PD, MSA, and PSP with high diagnostic accuracy as well as better sensitivity and specificity. However, no study has combined these potentially useful biomarkers. Thus, firstly, we employed QSM to compare iron deposition in the deep gray nucleus (including CN and DN) underlying the pathophysiology in patients with PSP, MSA, and PD to explore whether QSM can discriminate them; Secondly, we combined QSM and plasma NfL levels to determine whether the combination of these measures could improve accuracy in distinguishing PD from MSA and PSP with high sensitivity and specificity.

## Materials and methods

### Participants

Participants included 47 PD, 28 MSA, 18 PSP, and 28 age- and sex-matched healthy controls (HC). Among them, blood samples of 22 HC, 47 PD, 18 MSA, and 14 PSP patients were collected. All participants were recruited from the Outpatient and Inpatient Department of Guangdong Neuroscience Institute, Guangdong Provincial People’s Hospital, between April 2018 and June 2020. Participants in this study signed informed consent prior to the experiment. This case–control study was performed in accordance with the ethical standards of the Declaration of Helsinki and approved by the Ethics Committees of Guangdong Provincial People’s Hospital (No. GDREC2018338H [R1]).

The PD patients enrolled in this study were clinically established, which was made based on Movement Disorder Society (MDS) clinical criteria (Postuma et al., 2015). Probable MSA and PSP patients were diagnosed according to previously established criteria (Gilman et al., 2008; Jabbari et al., 2020). The part III of the MDS Unified Parkinson’s Disease Rating Scales (MDS-UPDRS III) and the Hoehn and Yahr (H-Y) scale in the off state was used to evaluate the severity of parkinsonian disorders. The Montreal Cognitive Assessment (MoCA) was used to assess the cognitive function of all participants. Patients were not taking any antiparkinsonian medications for at least 12 h before assessments. Any participants who suffered from other neurological diseases, tumors, neuroinflammation diseases, etc., were excluded. The demographic and clinical data are summarized in Table 1.

**TABLE 1:** Demographic and clinical characteristics.

	HC	PD	MSA	PSP	*P*
*n*	28 (22)	47 (47)	28 (18)	18 (14)	
Age	63.00 (20.00)	64.00 (10.00)	61.50 (12.00)	67.50 (9.00)	0.256
Gender, *n* Females (% female)	17 (60.71%)	21 (44.68%)	15 (53.57%)	7 (38.89%)	0.475
Education (years)	12.00 (7.00)	10.00 (6.00)	8.00 (4.00)	9.00 (5.50)	0.029
Disease duration		2.00 (3.00)	2.00 (2.00)	2.00 (3.25)	0.640
H-Y		2.00 (0.50)	2.50 (0.88)	3.00 (0.75)	0.001
MDS-UPDRS III		32.00 (19.00)	30.50 (13.25)	36.00 (20.50)	0.232
MoCA	27 (3.00)	20.00 (9.00)	18.00 (7.30)	16.00 (9.80)	<0.001
Follow-up time after MRI examination (months)		18.87 (18.13)	15.22 (10.25)	12.08 (10.79)	0.141

Data are presented with median (interquartile range), or as number and percentage. HC, healthy control; PD, Parkinson’s disease; MSA, multiple system atrophy; PSP, progressive supranuclear palsy; H-Y, Hoehn and Yahr scale; MoCA, Montreal Cognitive Assessment; MDS-UPDRS-III, part III of the Movement Disorder society-sponsored Revision of the Unified Parkinson’s Disease Rating Scale; MRI, magnetic resonance imaging.

### Neuroimaging

#### Brain magnetic resonance imaging acquisition

Magnetic resonance imaging (MRI) data acquisition and post-processing were described previously (Chen et al., 2020). A 3.0 T MRI system (GE Healthcare, Fairfield, United States) was used. A 3D-enhanced gradient echo SWAN sequence was performed with 13 spaced echoes (repetition time/echo time = 89.2/2.6–36.4 ms, flip angle = 30°, field of view = 240 mm × 240 mm, slice thickness = 3 mm, number of slices = 72, voxel size = 0.75 mm × 1.07 mm × 3 mm, bandwidth = 62.5 Hz/pixel, and matrix size = 320 × 224). For each patient with MSA and PSP, the presence of specific MR signs on conventional sequences was recorded blindly by neuroradiologist.

#### Quantitative susceptibility mapping reconstruction

Quantitative susceptibility mapping reconstruction was performed by MATLAB software (version R2016b) as described in previous studies (Chen et al., 2020; Sun et al., 2020). The code conversion mainly refers to the method provided in a tissue magnetic marker resonance solution using the morphology-enabled dipole inversion (MEDI) (Wang and Liu, 2015). Briefly, the QSM image was obtained by unwrapping the phase image, removing the background field, and reconstructing the image through the MEDI algorithm. And the MEDI algorithm was used to reconstruct QSM images with the following parameters: cg_max_iter = 100, cg_tol = 0.01, max_iter = 10, tol_norm_ratio = 0.1, lambda = 900, lambda CSF = 10, and merit = 1.

#### Regions of interest analysis

**FIGURE 1:**
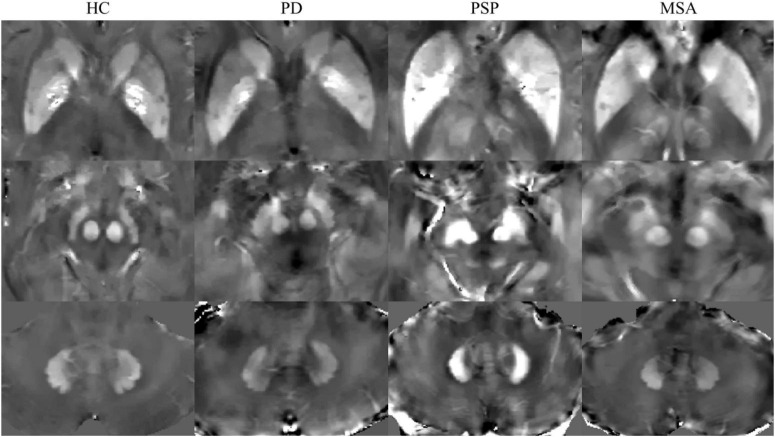
Representative quantitative susceptibility mapping (QSM) images of HC, PD, MSA, and PSP. HC, healthy control; PD, Parkinson’s disease; PSP, progressive supranuclear palsy; MSA, multiple system atrophy.

The susceptibility maps were obtained by ITK-SNAP3.6.0, and ROIs were manually segmented by two experienced neurologists who were blinded to the diagnosis of the participants, as described in our previous study (Chen et al., 2020). Six ROIs were selected, including the bilateral GP, PUT, CN, RN, SN, and DN. The unit of susceptibility values measured on QSM was expressed as parts per billion (ppb). The sums of the susceptibility values in the bilateral regions were used for the statistical analysis. To investigate the reproducibility and reliability of the QSM data, neurologist 1 responsible for the ROI analysis remeasured the nuclei 6 months later. Representative QSM images of ROIs in participants are shown in Figure 1.

### Plasma collection and analysis

Blood samples were collected using ethylene diamine tetraacetic acid anticoagulation tubes with an empty stomach in the morning, and centrifuged at 1,500 × *g* for 10 min at 4°C within 1 h. The supernatant was extracted and stored in an −80°C freezer. Plasma samples were thawed on ice and centrifuged at 10,000 × *g* for 5 min before further processing. Plasma NfL was quantified using ultrasensitive Simoa technology (Quanterix, Billerica, MA, United States) on the automated Simoa HD-X platform (GBIO, Hangzhou, China) per the manufacturer’s instructions. NF-light assay (Cat No: 103186) kits were purchased from Quanterix and used accordingly. Plasma samples were diluted at a 1:4 ratio before measurement. Calibrators, quality controls, and samples were measured in duplicate. One patient with PD and one patient with HC were excluded because of coefficient of variance (CV) > 15%. The assays were performed using kits with the same lot number. Operators were blinded to participant disease status.

### Statistical analysis

Statistical analysis was performed using SPSS Statistics version 20.0 (IBM SPSS Statistics, Armonk, NY, United States). Shapiro-Wilk test was used to test normality of the data. One-way ANOVA was used to compare the difference in normal continuous variables; otherwise, the Kruskal–Wallis *H*-test was used for data that did not conform to a normal distribution. The possible difference in sex distribution between groups was evaluated using the χ^2^ test. Furthermore, *post hoc* tests with a Bonferroni correction for multiple comparisons were performed, and corrected *P*-values are presented. Generalized linear models were used to compare the difference of QSM values between the groups, and age and sex were used as covariates, then followed by pairwise comparison. The area under the curve (AUC) of the receiver operating characteristic (ROC) curve was used to evaluate diagnostic accuracy of susceptibility values of ROIs and blood NfL for differentiating the groups (such as PSP vs. PD, MSA vs. PD, MSA vs. PSP, PSP vs. HC, MSA vs. HC, and PD vs. HC). To combine serum NfL and susceptibility values of the ROIs, binary logistic regression was first conducted, followed by ROC curves, and the AUC of ROC was used to evaluate diagnostic performance. The logistic regression model was designed to predict a transformation of the response variable logit (p), which was to estimate the probability of Parkinsonian disorders in relation to the QSM and blood NfL values. And the best cutoff regarding sensitivity and specificity was calculated by maximizing the Youden’s index. A corrected *P*-value < 0.05 was considered to be statistically significant.

## Results

### Demographic and clinical characteristics

There were no differences in age (*P* = 0.256), sex distribution (*P* = 0.475), disease duration (*P* = 0.640), or MDS-UPDRS III scores (*P* = 0.232) between groups (Table 1). Hoehn and Yahr stage was significantly different between groups, with the MSA and PSP patients having higher scores than the PD patients. Furthermore, the MSA and PSP groups had lower MoCA scores than the PD and HC groups (*P* < 0.001).

### Conventional imaging results

Abnormalities on conventional MRI signs in patients with MSA and PSP were recorded as follows: hot cross bun signs in 25% MSA, pons atrophy was observed in 32% of MSA, cerebellum atrophy in 46% of MSA, putaminal atrophy in 11% of MSA, and hummingbird sign or morning glory flower sign in 50% of PSP.

### Reproducibility of quantitative susceptibility mapping measurements

The intraclass correlation coefficient (ICC) for intra-rater agreement of measurements of deep gray nucleus were excellent; it was 0.993 for CN, 0.997 for PUT, 0.987 for GP, 0.996 for SN, 0.996 for RN, and 0.987 for DN, and all *P* < 0.001. The high ICC was observed in the inter-rater measurements of deep gray nucleus; it was 0.958 for CN, 0.987 for PUT, 0.985 for GP, 0.937 for SN, 0.994 for RN, and 0.967 for DN, and all *P* < 0.001.

### Differences in susceptibility values in the deep gray matter and the receiver operating characteristic analysis

**FIGURE 2:**
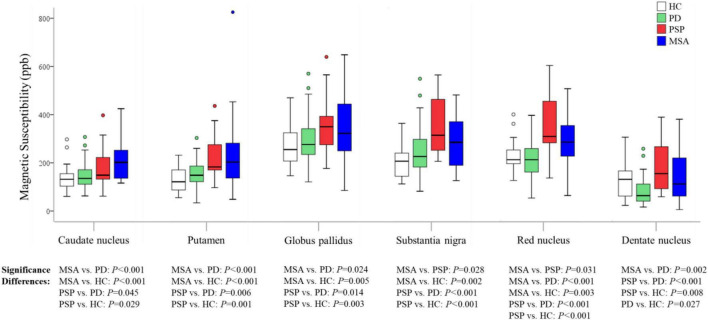
Group comparisons of susceptibility values in the regions of interest. The *P-*values are corrected for multiple comparisons. HC, healthy control; PD, Parkinson’s disease; PSP, progressive supranuclear palsy; MSA, multiple system atrophy.

Generalized linear models were used to examine the group differences; age and gender were used as covariates. Susceptibility values for all ROIs considered in the study are shown in Figure 2 and Table 2. We found highly significant differences in pairwise comparisons between the studied groups in each of the ROIs.

**TABLE 2:** Comparison of susceptibility values of all ROIs (ppb) and serum NfL levels (pg/ml) in HC, PD, MSA, and PSP.

	HC	PD	MSA	PSP	*P*
Caudate nucleus	131.61 (55.43)	135.26 (61.84)	201.92 (120.23)	148.64 (94.82)	<0.001
Putamen	121.36 (85.11)	148.20 (68.95)	203.24 (149.05)	182.83 (117.93)	<0.001
Globus pallidus	255.01 (118.61)	276.22 (114.11)	322.34 (201.26)	349.79 (155.05)	0.003
Substantia nigra	206.69 (98.21)	226.47 (116.74)	314.41 (222.11)	285.15 (190.32)	<0.001
Red nucleus	212.86 (61.40)	212.85 (108.99)	286.15 (135.82)	309.60 (180.79)	<0.001
Dentate nucleus	131.37 (112.77)	63.91 (73.05)	112.14 (160.50)	155.17 (182.86)	<0.001
NfL	9.95 (5.72)	16.32 (9.16)	33.14 (28.10)	24.74 (12.39)	<0.001

Data are presented with median (interquartile range). HC, healthy control; PD, Parkinson’s disease; MSA, multiple system atrophy; PSP, progressive supranuclear palsy; NfL, neurofilament light chain. Generalized liner models were used to compare the difference of QSM values between the groups with age and sex were used as covariates, and P-values of comparison between groups were provided in this table.

In MSA patients, susceptibility values in CN (*P* < 0.001), PUT (*P* < 0.001), GP (*P* = 0.024), RN (*P* < 0.001), and DN (*P* = 0.002) were found to be higher than in PD patients, with increased susceptibility values in CN (*P* < 0.001), PUT (*P* < 0.001), GP (*P* = 0.005), SN (*P* = 0.002), and RN (*P* = 0.003) than in HC, and susceptibility values in SN (*P* = 0.028), RN (*P* = 0.031) were found to be higher than in PSP patients.

In PSP patients, susceptibility values in CN (*P* = 0.045), PUT (*P* = 0.006), GP (*P* = 0.014), SN (*P* < 0.001), RN (*P* < 0.001), and DN (*P* < 0.001) were found to be higher than in PD patients, and susceptibility values in CN (*P* = 0.029), PUT (*P* = 0.001), GP (*P* = 0.003), SN (*P* < 0.001), RN (*P* < 0.001), and DN (*P* = 0.008) were found to be higher than in HC.

**FIGURE 3:**
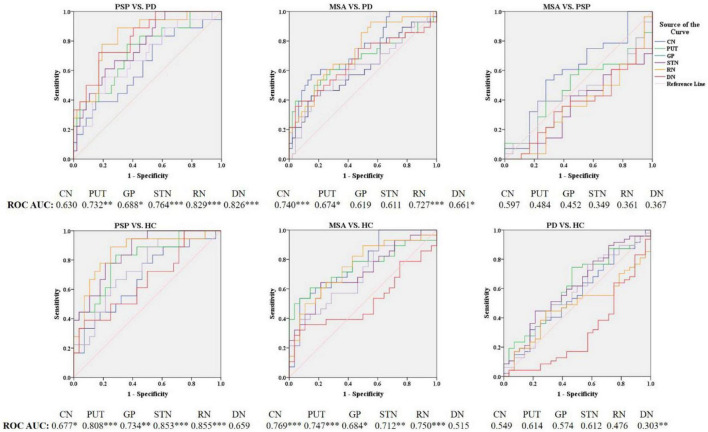
Receiver operating characteristic (ROC) curves showing comparisons in the regions of interest between parkinsonian disorders and HC. *P < 0.05, ^**^P ≤ 0.01, ^***^P ≤ 0.001. CN, caudate nucleus; PUT, putamen; GP, globus pallidus; SN, substantia nigra; RN, red nucleus; DN, dentate nucleus.

In PD patients, susceptibility value in DN (*P* = 0.027) was lower than in HC. We performed an exploratory analysis of diagnostic accuracy of QSM measures using the same data set. The diagnostic accuracy of susceptibility values in the different ROIs in diagnosing MSA, PSP, and PD was determined using the area under the curve (AUC). As shown in Figure 3 and Table 3, the most promising biomarker for differentiating PSP from PD was susceptibility level in the RN, with an AUC of 0.829, the sensitivity was 88.9%, and specificity was 70.2%. Susceptibility level in the CN had the best accuracy in differentiating MSA from PD, with an AUC of 0.740, the sensitivity was 57.1%, and specificity was 85.1%. Regarding the separation from controls, the best test performances were achieved in the RN with an AUC of 0.855 for PSP and in the CN with an AUC of 0.769 for MSA. However, in differentiating HC from PD, the best accuracy was in the DN, with an AUC of 0.697.

**TABLE 3:** Significant differences of diagnostic accuracy in the comparison with HC, PD, MSA, and PSP.

	AUC	CI (95%)	Sensitivity	Specificity	Cutoff
**MSA vs. PD**					
Caudate nucleus	0.740	0.624–0.856	57.1%	85.1%	191.837
Putamen	0.674	0.537–0.811	39.3%	95.7%	241.208
Red nucleus	0.727	0.609–0.845	92.9%	44.7%	199.38
Dentate nucleus	0.661	0.523–0.799	39.3%	91.5%	160.478
NfL	0.877	0.758–0.996	83.3%	91.5%	24.410
Caudate nucleus + NfL	0.872	0.748–0.997	88.9%	87.2%	0.278
**PSP vs. PD**					
Putamen	0.732	0.595–0.868	77.8%	63.8%	167.524
Globus pallidus	0.688	0.543–0.833	55.6%	78.7%	347.872
Substantia nigra	0.764	0.642–0.885	61.1%	78.7%	300.794
Red nucleus	0.829	0.720–0.938	88.9%	70.2%	250.882
Dentate nucleus	0.826	0.721–0.932	72.2%	83.0%	122.044
NfL	0.810	0.687–0.933	64.3%	89.4%	23.715
Red nucleus + NfL	0.904	0.830–0.978	92.9%	83.0%	0.247
**MSA vs. HC**					
Caudate nucleus	0.769	0.646–0.892	53.6%	92.9%	196.711
Putamen	0.747	0.614–0.881	60.7%	85.7%	183.312
Globus pallidus	0.684	0.544–0.823	39.3%	92.9%	410.268
Substantia nigra	0.712	0.576–0.847	64.3%	78.6%	242.786
Red nucleus	0.750	0.621–0.879	89.3%	50.0%	210.584
NfL	0.934	0.855–1.000	88.9%	95.5%	20.60
Caudate nucleus + NfL	0.927	0.841–1.000	88.9%	95.5%	0.545
**PSP vs. HC**					
Caudate nucleus	0.677	0.536–0.817	77.8%	53.6%	131.965
Putamen	0.808	0.678–0.937	77.8%	75.0%	169.312
Globus pallidus	0.734	0.587–0.881	88.9%	50.0%	250.579
Substantia nigra	0.853	0.747–0.959	77.8%	78.6%	46.714
Red nucleus	0.855	0.736–0.974	88.9%	75%	249.052
NfL	0.929	0.841–1.000	100.0%	77.3%	13.535
Red nucleus + NfL	0.994	0.976–1.000	92.9%	100.0%	0.776
**PD vs. HC**					
Dentate nucleus	0.697	0.570–0.823	57.1%	83.0%	120.359
NfL	0.764	0.640–0.888	83%	63.6%	10.605
Dentate nucleus + NfL	0.843	0.728–0.959	87.%	77.3%	0.599

HC, healthy control; PD, Parkinson’s disease; MSA, multiple system atrophy; PSP, progressive supranuclear palsy; NfL, neurofilament light chain; AUC, area under the curve; CI, confidence interval.

### Differences in neurofilament light levels between groups and the receiver operating characteristic analysis

**FIGURE 4:**
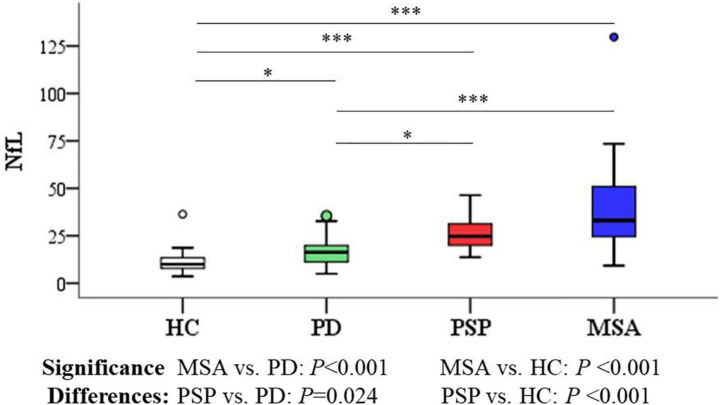
Group comparisons of plasma NfL between parkinsonian disorders and HC. The *P*-values are corrected for multiple comparisons. *P < 0.05, ^***^P ≤ 0.001. HC, healthy control; PD, Parkinson’s disease; PSP, progressive supranuclear palsy; MSA, multiple system atrophy.

**FIGURE 5:**
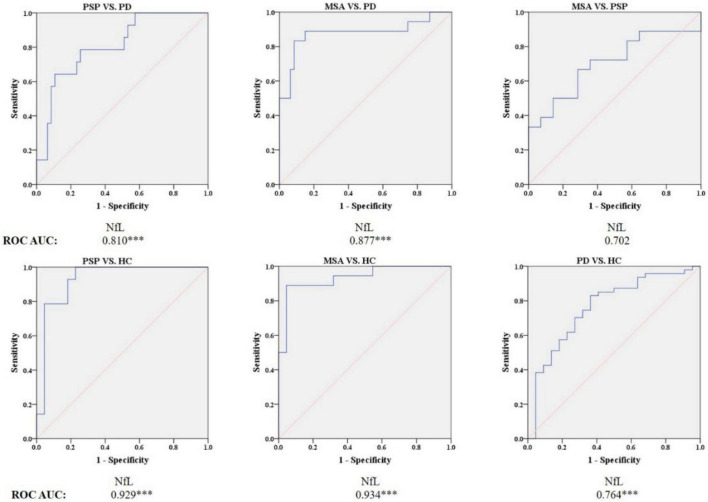
Receiver operating characteristic (ROC) curves showing comparisons in plasma NfL between parkinsonian disorders and HC. ^***^P ≤ 0.001. HC, healthy control; PD, Parkinson’s disease; PSP, progressive supranuclear palsy; MSA, multiple system atrophy.

As shown in Figures 4, 5 and Table 3, the levels of blood NfL were distinctly increased in PSP and MSA patients compared to HC (PSP, *P* < 0.001; MSA, *P* < 0.001) and patients with PD (PSP, *P* = 0.024; MSA, *P* < 0.001). The PD group showed a higher level of NfL than the HC group (*P* = 0.034). There was no significant difference between PSP and MSA.

Blood NfL levels distinguished patients with MSA and PSP from patients with PD with relatively high accuracy (PSP, AUC: 0.810, MSA, AUC: 0.877). The sensitivity was 64.3% and specificity was 89.4% at a cutoff value of 23.715 pg/ml for distinguishing PSP from PD, and the sensitivity was 83.3% and specificity was 91.5% at a cutoff value of 24.41 pg/ml for differentiating MSA from PD. Similarly, blood NfL levels could separate patients with PSP, MSA, and PD from HC (PSP, AUC: 0.929, MSA, AUC: 0.934, PD, AUC: 0.764).

### Receiver operating characteristic analysis of the combination of quantitative susceptibility mapping and neurofilament light levels

**FIGURE 6:**
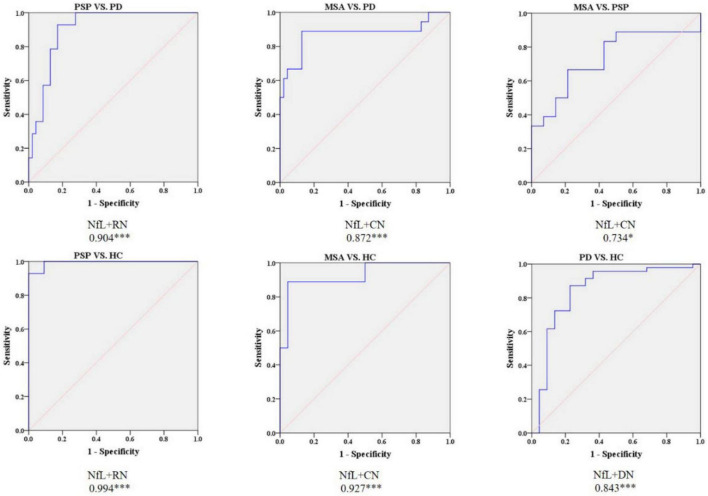
Receiver operating characteristic (ROC) curves showing comparisons in combining QSM and plasma NfL between parkinsonian disorders and HC. *P < 0.05, ^***^P ≤ 0.001. HC, healthy control; PD, Parkinson’s disease; PSP, progressive supranuclear palsy; MSA, multiple system atrophy.

According to the best diagnostic performance for distinguishing parkinsonian disorders, we combined susceptibility values in the RN and NfL levels to distinguish between PSP and PD patients as well as HC; CN values and NfL levels to discriminate MSA from PD patients and HC; and DN values and NfL levels to separate PD patients from HC. As shown in Figure 6 and Table 3, the diagnostic performance of combining susceptibility values in the RN and NfL levels (AUC: 0.904) was higher than that of the susceptibility value in the RN (AUC: 0.829) or blood NfL levels (AUC: 0.810) in the differentiation of PSP from PD patients, the sensitivity was 92.9% and specificity was 83.0% when the logit P is greater than or equal to 0.247. In addition, the diagnostic accuracy of combining susceptibility values in the CN and NfL levels (AUC: 0.872) was similar with blood NfL levels (AUC: 0.877) with sensitivity 88.9% and specificity 87.2% in separating MSA from PD patients, with a higher accuracy of combining susceptibility values in the DN and blood NfL levels (AUC: 0.843) than susceptibility values in the DN (AUC: 0.697) or blood NfL levels (AUC: 0.764) in differentiating PD from HC. However, there was no significant difference in combining QSM values and NfL levels to discriminate between MSA and PSP patients.

## Discussion

The main findings of this study show that the highest levels of susceptibility values in PSP patients were detected in the RN and in MSA patients were detected in CN compared with PD patients and HC. In addition, combining the susceptibility values in the RN and blood NfL levels clearly improved the diagnostic accuracy in the separation of PSP from PD while blood NfL level could accurately distinguish MSA from PD.

Conventional MRI signs of MSA and PSP showed high specificity but low sensitivity, giving a limited support to clinical diagnostic criteria (Mazzucchi et al., 2019), which is consistent with the study. The distinct subtypes of parkinsonian disorders are characterized by differences in susceptibility values and showed differences in iron accumulation in deep gray matter nuclei associated with these neurodegenerative disorders, thus providing a potential diagnostic biomarker (Ward et al., 2014). The highest susceptibility values in the PSP patients were detected in the RN, and increased susceptibility values were detected in the CN, PUT, GP, SN, and DN compared to PD patients, in accordance with previous studies (Mazzucchi et al., 2019). Recently, the majority of studies have found changes in the mesencephalic nucleus with an abnormal midbrain-pons area ratio and MR parkinsonism index (MRPI) measurement that has low sensitivity for discriminating PSP from PD (Whitwell et al., 2017). This is in line with pathological studies uncovering the abundance of neurofibrillary tangles and tufted astrocytes as a sign of neurodegeneration in this region (Hattori et al., 2003; Ebashi et al., 2019). Tau aggregates may hamper axonal transport and iron metabolism, thereby causing excessive iron accumulation in the mesencephalic nucleus (Boelmans et al., 2012). Indeed, a postmortem study confirmed that iron accumulation in the subcortical nucleus coincides with pathological lesions (Langkammer et al., 2010). This study suggested that iron dysregulation possibly contributes to the pathophysiology in the RN in PSP patients.

Iron accumulation in specific regions has been a pathological hallmark in MSA (Dexter et al., 1991). Unlike published studies found significantly increased iron content in PUT (Sjöström et al., 2017; Mazzucchi et al., 2019), our study found that the highest iron content was recorded in CN in the MSA patients, which was consistent with neuropathology changes with greater inclusion pathology in the CN in patients with MSA. Another explanation may be more than half of MSA patients enrolled in this study were MSA with prominent cerebellar symptoms and iron-related degeneration in the PUT may be more specific for MSA with predominantly parkinsonism. In addition, one of the studies did not include the CN in analyses to distinguish between APDs and PD (Sjöström et al., 2017). Consistent results have also been found in pathological studies, which suggest the presence of α-synuclein in glial cytoplasmic inclusions within striatonigral systems in MSA patients (Ahmed et al., 2012). Some functional imaging also showed widespread microglial activation in CN in patients with MSA, it was assumed that more iron deposit in CN (Kübler et al., 2019). At present, it is still debated whether iron deposition is the initial factor leading to neurodegeneration or whether iron deposition aggravates disease progression. When a study noted that iron significantly accelerated the formation of α-synuclein fibril (Uversky et al., 2001). Lee et al. (2019) suggested that iron accumulation in the PUT of MSA patients was likely a secondary byproduct of neurodegeneration. Thus, more prospective and interventional studies are needed to determine the role of iron deposition in neurodegenerative diseases. Unlike two previous studies that found the RN had higher diagnostic performance in distinguishing PSP and MSA patients (Sjöström et al., 2017; Mazzucchi et al., 2019), our study did not find any statistical significance. The relatively small sample size of previous studies may be an explanation. PD patients had lower susceptibility values than HC in the DN, which is consistent with a previous study that enrolled primarily predominantly akinetic-rigid PD (PD-AR) (Acosta-Cabronero et al., 2017); thus, decreased QSM appears to be concordant with those recent studies that has hinted at susceptibility value in DN being associated with phenotypic differences in that tremor-predominant PD (PD-TD) cases had increased values, whereas there was a trend to reduce susceptibility in PD-AR (He et al., 2017; Chen et al., 2020). Therefore, our study revealed the distinct iron deposition patterns of parkinsonian disorders, and QSM may be a potential imaging biomarker of pathophysiologic changes for differentiating PD from APDs. However, we need higher diagnostic accuracy of the biomarkers for applications in clinical practice.

Consistent with previous studies (Hansson et al., 2017; Lin et al., 2019), we found blood levels of NfL were greatly increased in patients with MSA and PSP compared with PD and HC patients. Blood NfL levels could be a potential biomarker for the differential diagnosis of PD and APDs. Furthermore, unlike previous studies, we found increased NfL levels in patients with PD compared with HC, which can possibly be explained by the fact that PD patients in our study had a lower MoCA score. Higher levels of NfL were correlated with lower MoCA scores (Lin et al., 2019; Lerche et al., 2020). Similarly, Zhang et al. (2022) revealed that blood NfL was a reliable biomarker for the disease severity of MSA and monitoring the progression of MSA. Consistent with this study, NfL was a potential biomarker for differentiating MSA from PD. NfL primarily is located in long myelinated axons, elevated NfL should most closely reflect white-matter changes (Khalil et al., 2018). Recently, numerous studies have found reduced fractional anisotropy in subcortical white matter in patients with MSA and PSP, reflecting microstructural white matter impairment in neurodegeneration (Krismer et al., 2021). Simultaneously, there are other longitudinal analyses revealed that elevated NfL in aging reflects brain white-matter alterations (Benedet et al., 2020; Nyberg et al., 2020). Taken together, blood NfL levels could be used as an indicator that reflects white matter axonal damage in the brain (Lin et al., 2019).

Many studies have indicated gray matter and white matter impairments in patients with parkinsonian disorders. Dash et al. (2019) pointed out that early changes, including loss of gray matter in the cerebellum and subcallosal gyrus with widespread involvement of supratentorial and infratentorial white matter fibers, were observed in patients with APDs. These gray matter and white matter changes were also found in PD and PSP patients (Agosta et al., 2018). These changes indicated that patients with parkinsonian disorders have comprehensive cerebral impairments that damage not only the deep nuclei but also white matter fibers. Until now, the mechanisms underlying these changes in parkinsonian disorders have remained unknown. Several studies have reported that α-synuclein and neurofibrillary tangles accumulate in both neurons and oligodendrocytes (Wong and Krainc, 2017), which may be a possible mechanism. At present, there is no single biomarker that reflects damage to both gray and white matter. Therefore, considering that QSM is an indicator of deep gray matter nuclei with NfL levels reflecting white matter impairment; we presume that the combination of QSM of deep gray matter nuclei and NfL levels possibly improves diagnostic performance in differentiating PSP from PD. To the best of our knowledge, our findings have never been reported, where combining the susceptibility values in the RN and NfL levels has higher diagnostic accuracy for distinguishing PSP from PD patients with blood NfL levels for discriminating MSA from PD patients.

There are some limitations in this study. First, the major limitation of the study is that it is a case-control study with limited number of MSA and PSP patients and without external validation set and subtype analysis, which could result in over-estimating performance of these biomarkers. In addition, the study did not identify biomarkers to distinguish between MSA and PSP. Second, the diagnoses of these patients were carefully determined by neurologists and radiologists based on diagnostic criteria and neuroimaging findings, but were not pathologically confirmed. Therefore, there is a need to validate our findings in larger longitudinal cohorts with further postmortem cases, which will validate the sensitivity and specificity of QSM and NfL as an early diagnostic marker in parkinsonism. Third, cutoff of NfL in blood might vary depending on the disease severity and quantification techniques. It should also be noted that blood NfL is relatively low specificity and also abnormal in other conditions including nervous system diseases and renal disease (Gaetani et al., 2019; van der Plas et al., 2021). While these methods are promising, future studies are needed to substantiate their usefulness as biomarker in early parkinsonism. Fourth, automated delineation of the ROIs may allow for faster and more consistent analyses. Atlas-based autosegmentation for ROIs or novel positioning technologies should be considered in future research (Chen et al., 2020).

## Conclusion

In summary, the exploratory study indicates QSM may be a promising tool to quantitatively estimate iron deposition and reveal different patterns of iron accumulation in patients with PD, MSA, and PSP. The susceptibility values in the RN yielded the highest diagnostic performance for PSP, and the values in the CN obtained the best diagnostic accuracy for MSA. Furthermore, combining the susceptibility values in the RN and blood NfL levels clearly improved the diagnostic accuracy for PSP, and blood NfL levels has a high accuracy for the diagnosis of MSA.

## Data availability statement

The datasets used and analyzed during the current study are available from the corresponding author on reasonable request.

## Ethics statement

The studies involving human participants were reviewed and approved by the Ethics Committees of Guangdong Provincial People’s Hospital. The patients/participants provided their written informed consent to participate in this study.

## Author contributions

PZ, JC, TC, and YZ designed and organized this study. PZ, JC, TC, CH, YL, XL, ZC, and LW assisted with patient enrollment and data collection. PZ and JC were responsible for MRI image collection, processing, and data analysis. PZ and YZ prepared the first draft and participated in the revision of the manuscript. YZ supported this study financially. All authors contributed to the article and approved the submitted version.
